# Supplementary material for: Identification of Antimycobacterial Natural Products from a Library of Marine Invertebrate Extracts
Source: Medicines (Basel). 2022 Jan 28;9(2):9. doi: 10.3390/medicines9020009 (PMC8880526; doi:10.3390/medicines9020009)
Supplement: Supplementary file 1 [file medicines-09-00009-s001.zip › medicines-1548047-supplementary.pdf]

Supplementary Material

Article

Identification of antimycobacterial natural products from a library of marine invertebrate extracts

Kojo Sekyi Acquah[1,2], Denzil R. Beukes[3,*], Ronnett Seldon[4], Audrey Jordaan[5], Suthananda. N. Sunassee[1], Digby F. Warner[5,6] and David W. Gammon[1,*]

[1] Department of Chemistry, University of Cape Town, Rondebosch 7701, South Africa; ACQKOJ001@myuct.ac.za (K.S.A.); snsunassee@gmail.com (S.N.S.)
[2] Current address (K.S.A.): Division of Medicinal Chemistry, Department of Pharmaceutical Sciences, University of Connecticut, Storrs, CT 06269, USA
[3] School of Pharmacy, University of the Western Cape, Bellville 7535, South Africa
[4] Drug Discovery and Development Centre, Department of Chemistry, University of Cape Town, Cape Town 7700, South Africa; ronnett.seldon@uct.ac.za
[5] SAMRC/NHLS/UCT Molecular Mycobacteriology Research Unit & DST/NRF Centre of Excellence for Biomedical TB Research, Department of Pathology, Faculty of Health Sciences, University of Cape Town, Observatory 7925, South Africa; digby.warner@uct.ac.za (D.F.W); audrey.jordaan@uct.ac.za (A.J)
[6] Institute of Infectious Disease and Molecular Medicine, Faculty of Health Sciences, University of Cape Town, Observatory 7925, South Africa
[7] Wellcome Centre for Infectious Diseases Research in Africa, University of Cape Town, Rondebosch 7701, South Africa

List of Figures

Figure S1. HR-ESI-MS spectrum of heteronemin **1** 3
Figure S2. ^{1}H-NMR spectrum (600MHz, $CDCl_3$, 303K) of heteronemin **1** 4
Figure S3. ^{13}C-NMR spectrum (150MHz, $CDCl_3$, 303K) of heteronemin **1** 5
Figure S4. HSQC NMR spectrum (600MHz, $CDCl_3$, 303K) of heteronemin **1** 6
Figure S5. ^{1}H-^{1}H COSY spectrum (600MHz, $CDCl_3$, 303K) of heteronemin **1** 7
Figure S6. HMBC NMR spectrum (600MHz, $CDCl_3$, 303K) of heteronemin **1** 8
Figure S7. HR-ESI-MS spectrum of bengamide P **2** 9
Figure S8. ^{1}H-NMR spectrum (600MHz, $CDCl_3$, 303K) of bengamide P **2** 10
Figure S9. ^{13}C-NMR spectrum (150MHz, $CDCl_3$, 303K) of bengamide P **2** 11
Figure S10. ^{1}H-^{1}H COSY spectrum (600MHz, $CDCl_3$, 303K) of bengamide P **2** 12
Figure S11. HSQC NMR spectrum (600MHz, $CDCl_3$, 303K) of bengamide P **2** 13
Figure S12. HMBC NMR spectrum (600MHz, $CDCl_3$, 303K) of bengamide P **2** 14
Figure S13. HR-ESI-MS spectrum of bengamide Q **3** 15
Figure S14. ^{1}H-NMR spectrum (600MHz, $CDCl_3$, 303K) of bengamide Q **3** 16
Figure S15. ^{13}C-NMR spectrum (150MHz, $CDCl_3$, 303K) of bengamide Q **3** 17
Figure S16. HSQC NMR spectrum (600MHz, $CDCl_3$, 303K) bengamide Q **3** 18
Figure S17. ^{1}H-^{1}H COSY spectrum (600MHz, $CDCl_3$, 303K) of bengamide Q **3** 19
Figure S18. ^{1}H-^{1}H TOCSY spectrum (600MHz, $CDCl_3$, 303K) of bengamide Q **3** 20
Figure S19. HMBC NMR spectrum (600MHz, $CDCl_3$, 303K) of bengamide Q **3** 21
Figure S20. ^{1}H-NMR spectrum (600MHz, $CDCl_3$, 303K) of F107..22
Figure S21. ^{1}H-NMR spectrum (600MHz, $CDCl_3$, 303K) of F114..23
Figure S22. ^{1}H-NMR spectrum (600MHz, $CDCl_3$, 303K) of F130..24
Figure S23. MS/MS fragmentation pattern of bengamide R **4** (A) and bengamide S **5** (B) with fragment ions circled in red showing methylation is on the nitrogen of the caprolactam ring. 25

List of Table

Table S1. ^{13}C and ^{1}H chemical shifts of Heteronemin **1** isolated and that reported in literature. 26
Table S2. ^{13}C and ^{1}H chemical shifts of bengamide P **2** isolated and that reported in literature. 27
Table S3. ^{13}C and ^{1}H chemical shifts of bengamide Q **3** isolated and that reported in literature. 28
Table S4. Compounds isolated and tentatively identified in the molecular cluster of the crude extract (SS2) of the sponge Jaspis splendens with their corresponding masses (observed and calculated), molecular formulae (MF), and mass error (ID (Δ ppm)) 29

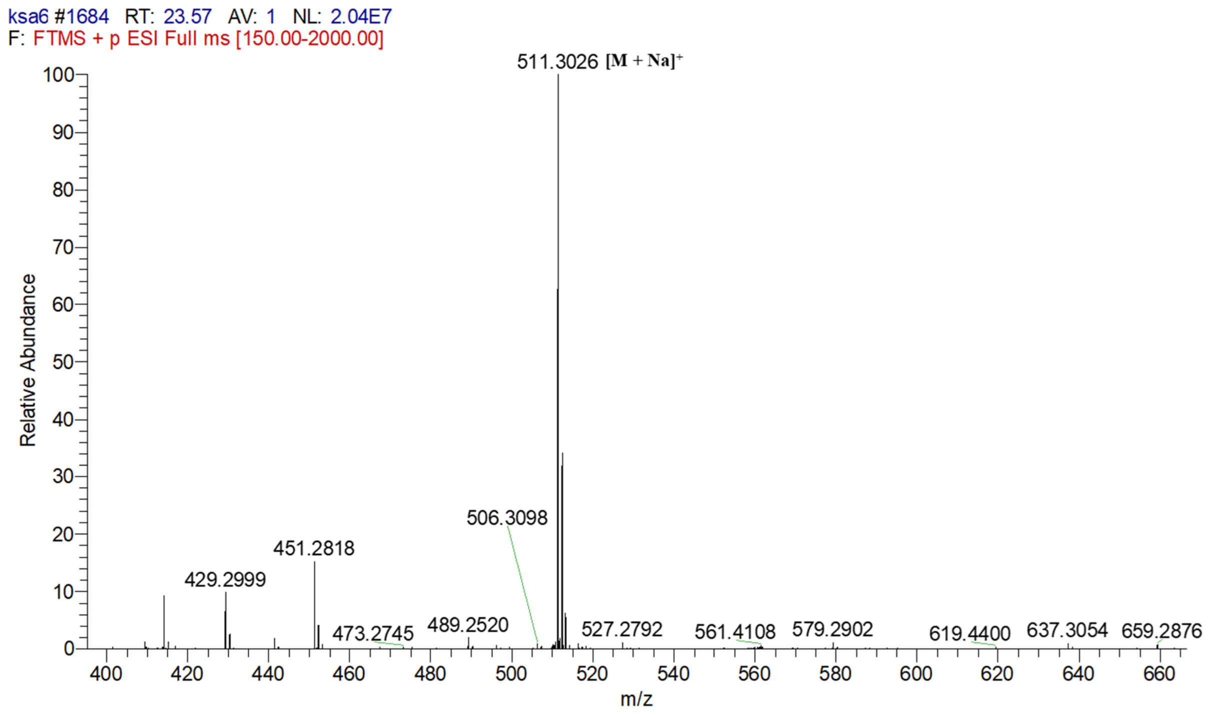

Figure S1. HR-ESI-MS spectrum of heteronemin **1**

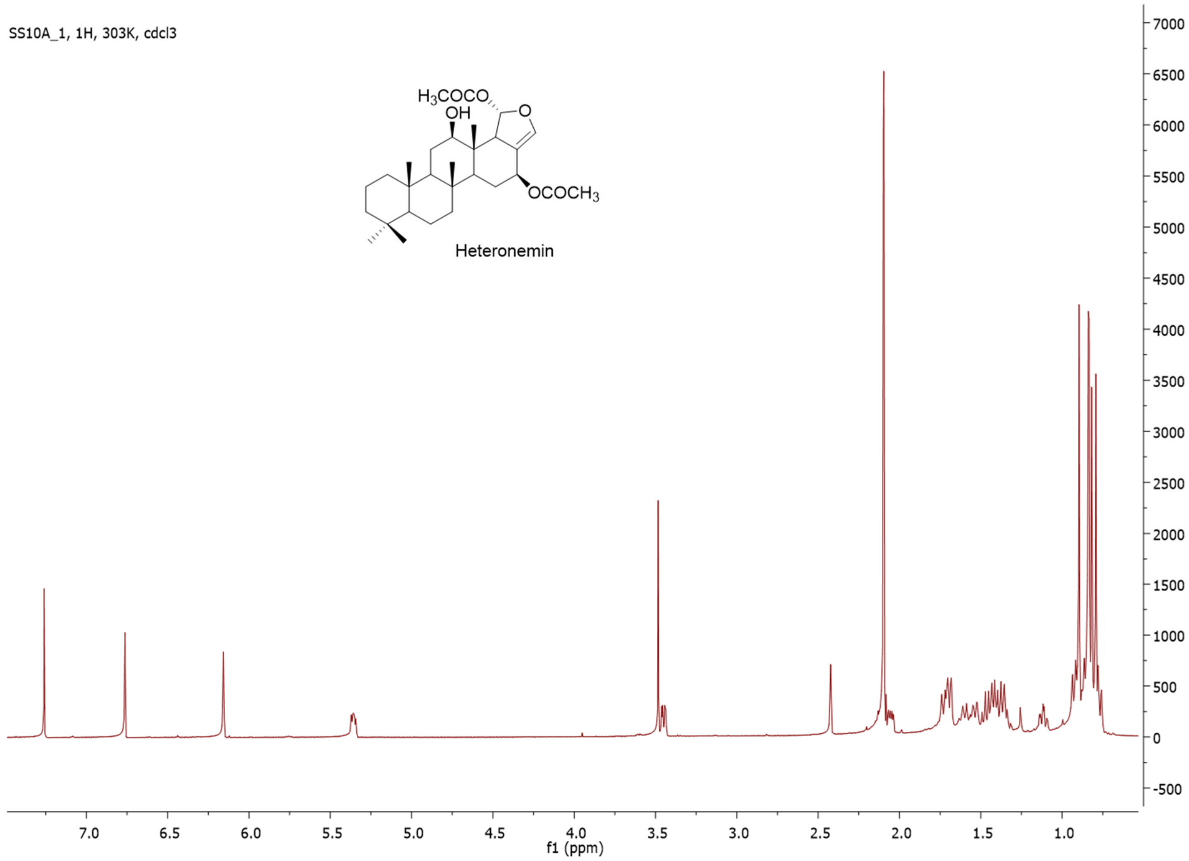

Figure S2. ^{1}H-NMR spectrum (600MHz, $CDCl_3$, 303K) of heteronemin **1**

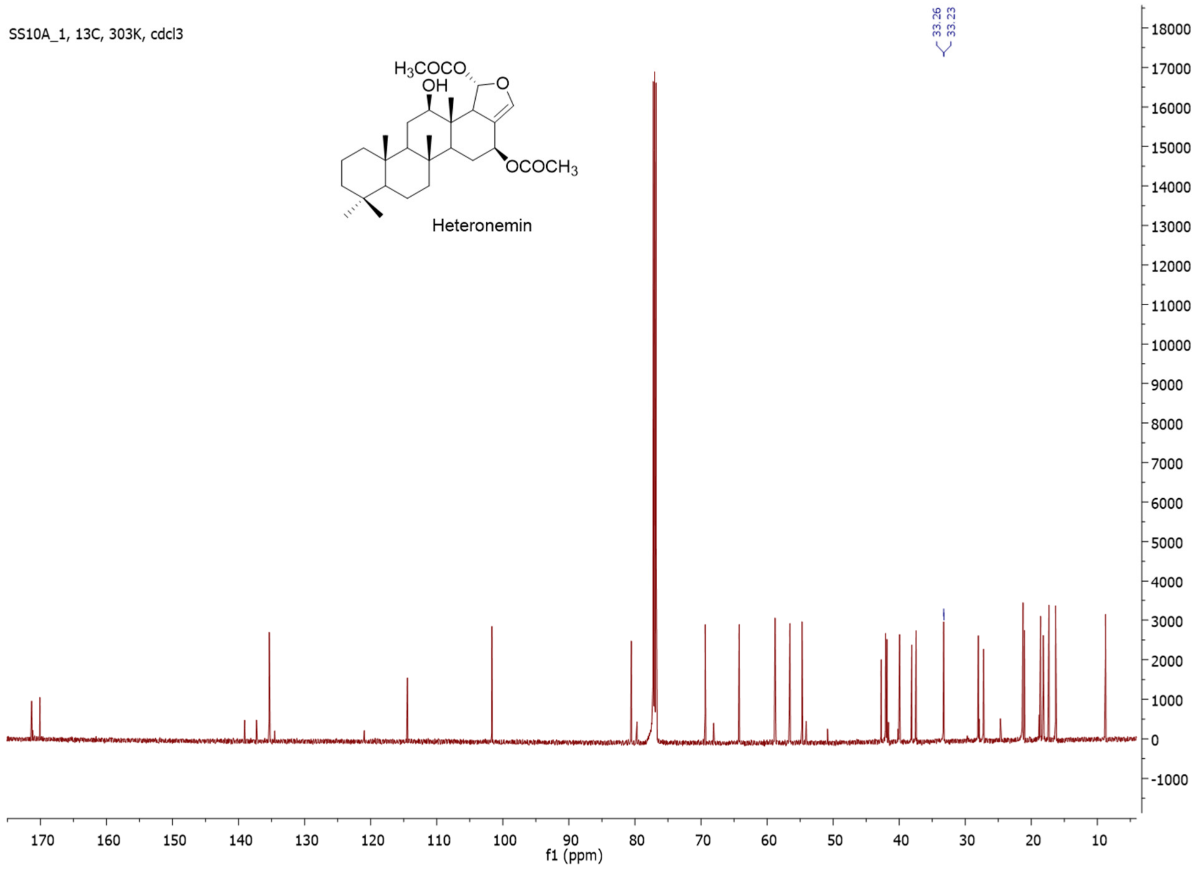

Figure S3. ^{13}C-NMR spectrum (150MHz, $CDCl_3$, 303K) of heteronemin **1**

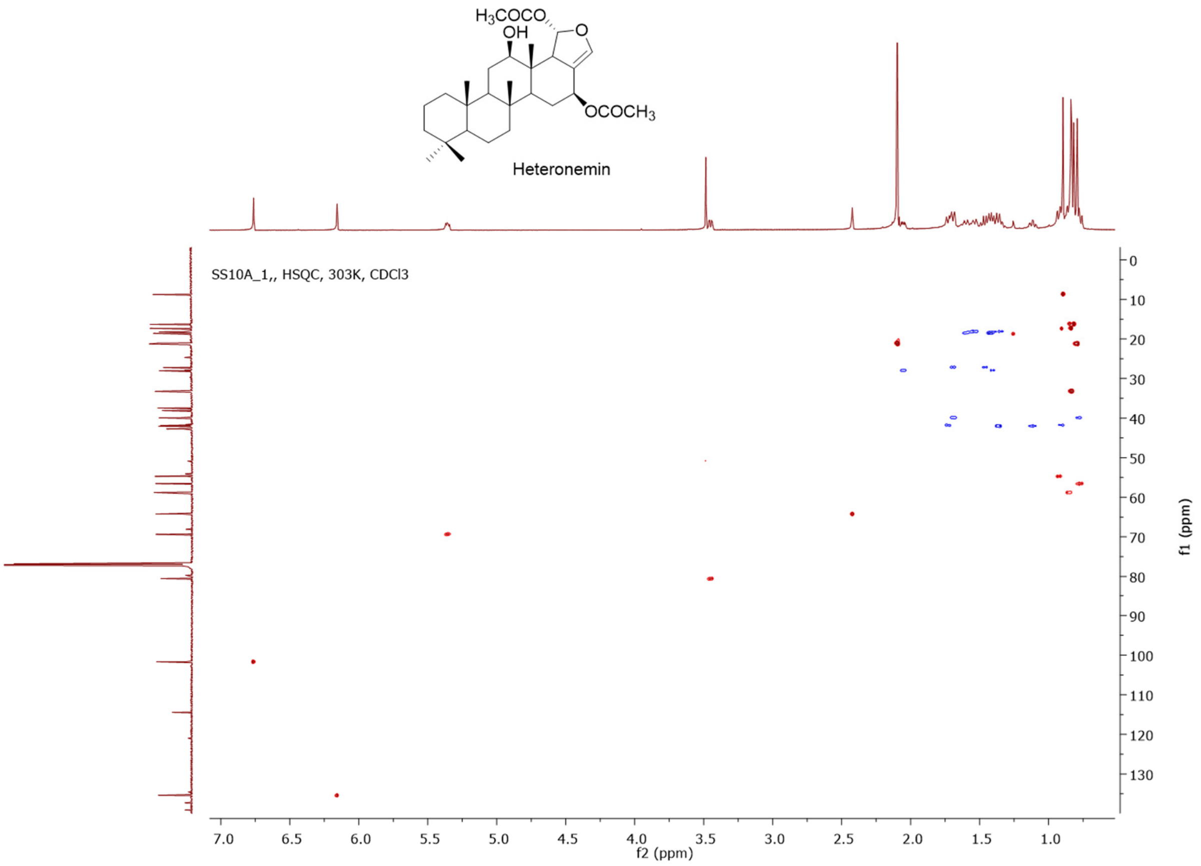

Figure S4. HSQC NMR spectrum (600MHz, $CDCl_3$, 303K) of heteronemin **1**

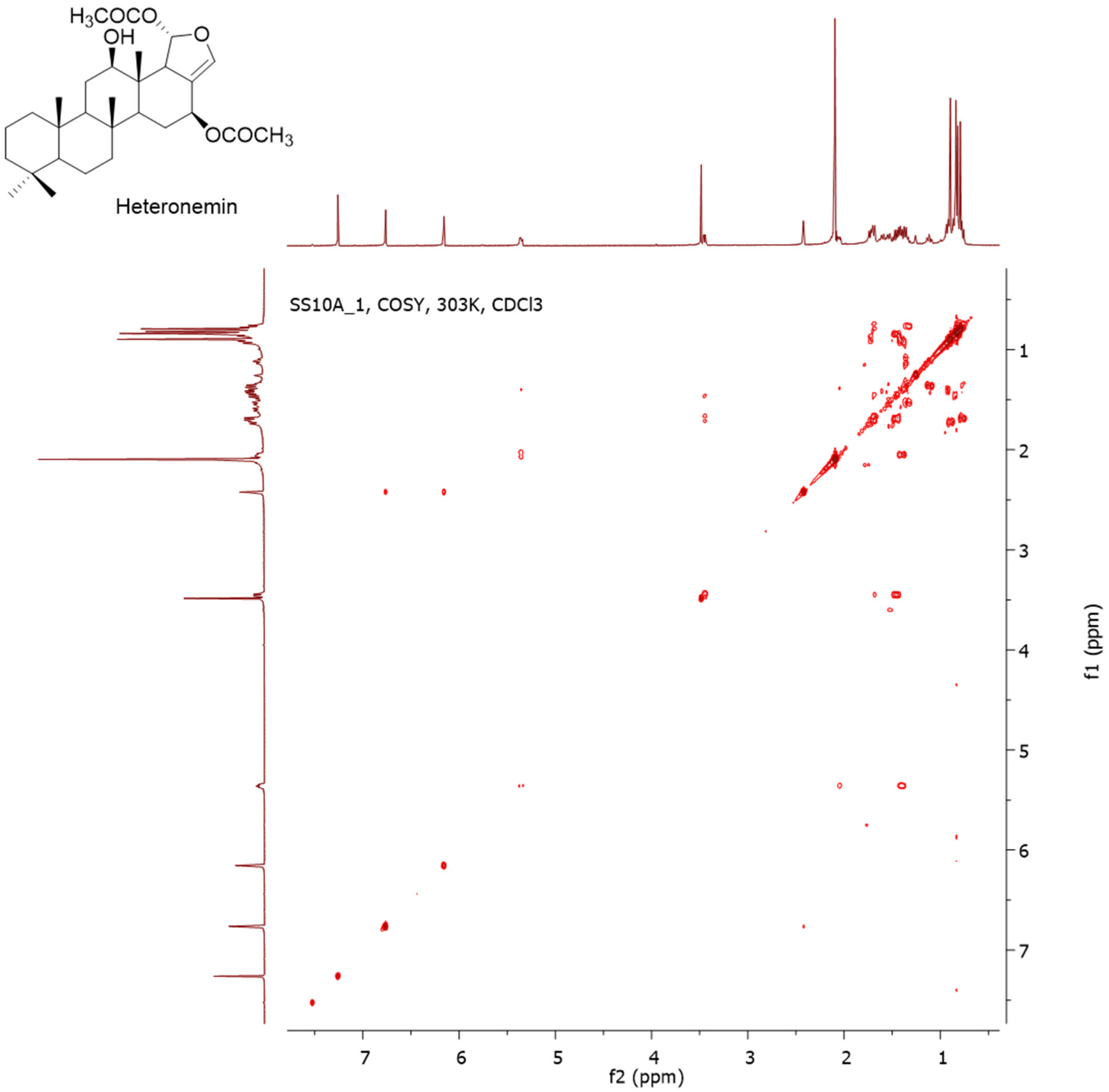

Figure S5. ^1H-^1H COSY spectrum (600MHz, $CDCl_3$, 303K) of heteronemin **1**

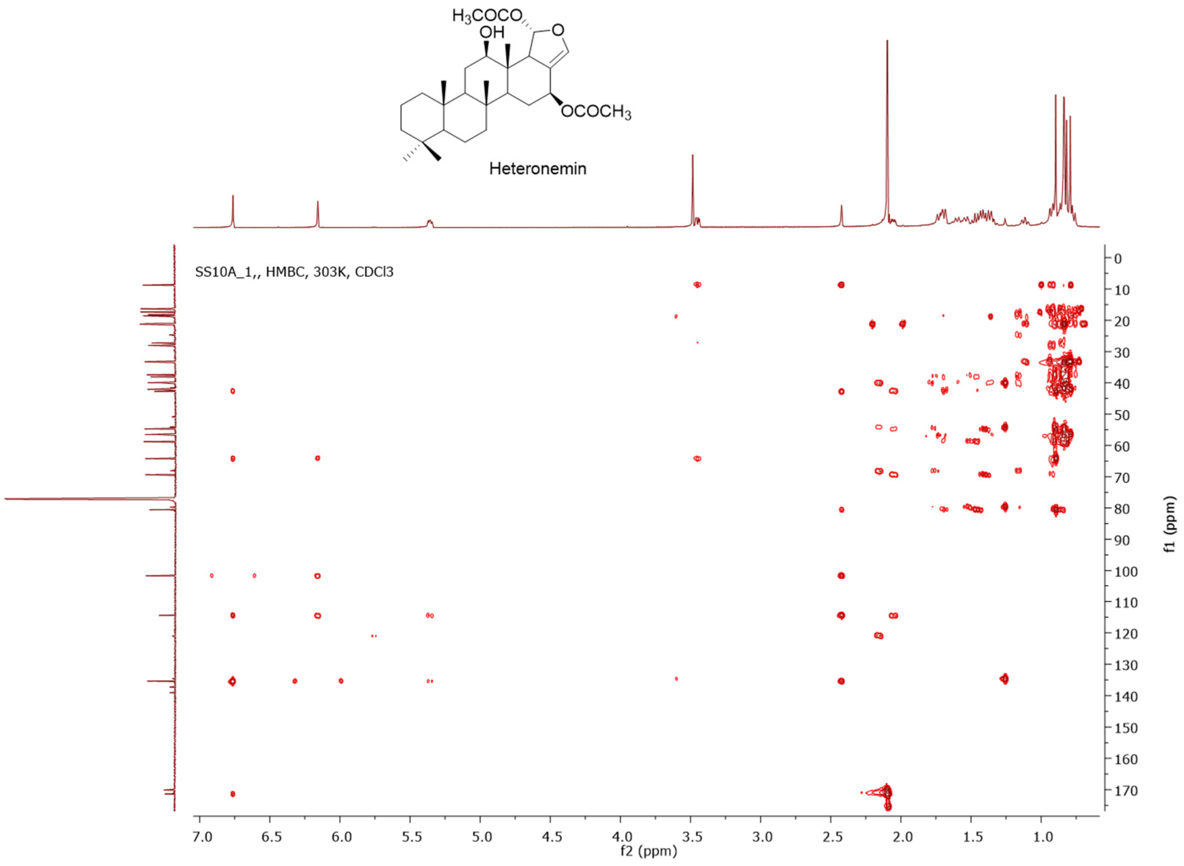

Figure S6. HMBC NMR spectrum (600MHz, $CDCl_3$, 303K) of heteronemin **1**

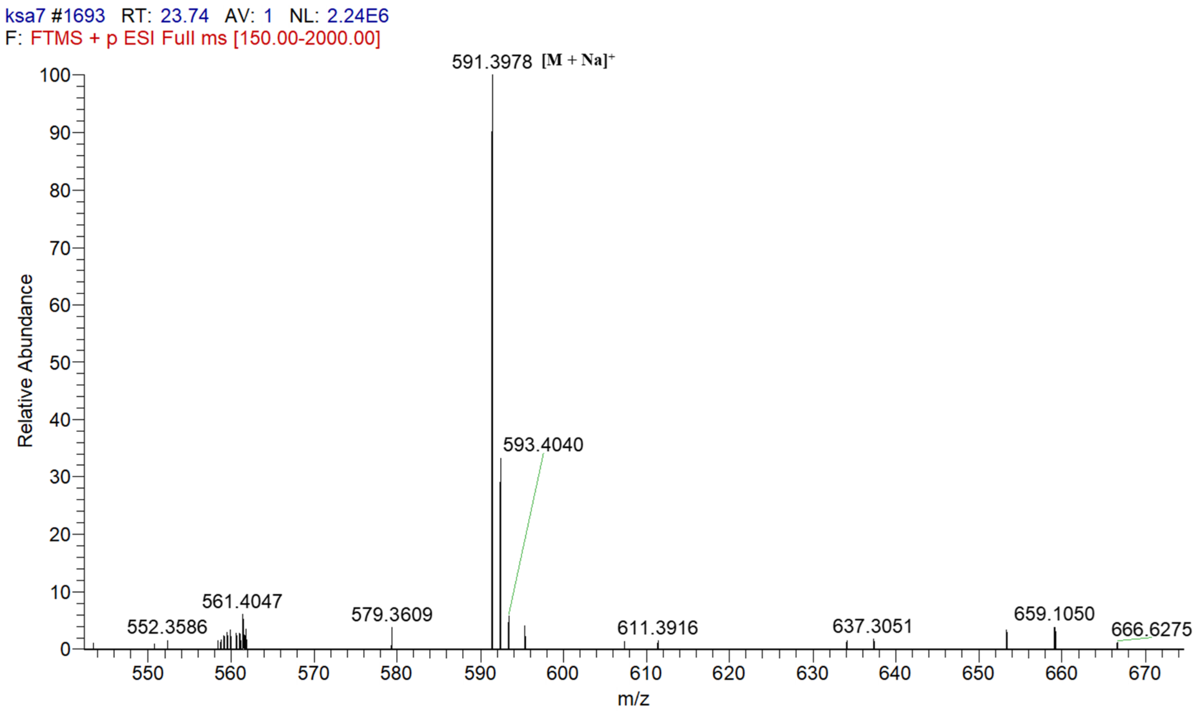

Figure S7. HR-ESI-MS spectrum of bengamide P **2**

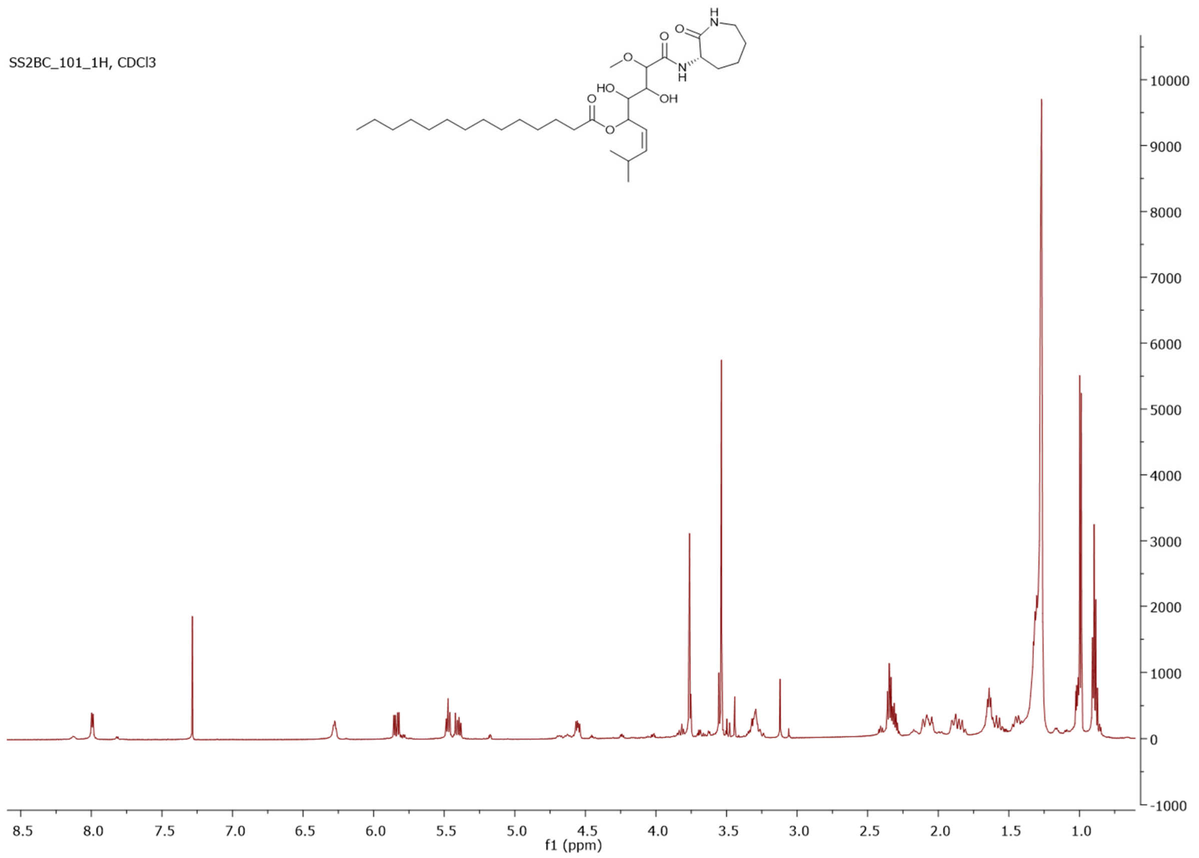

Figure S8. ^{1}H-NMR spectrum (600MHz, $CDCl_3$, 303K) of bengamide P **2**

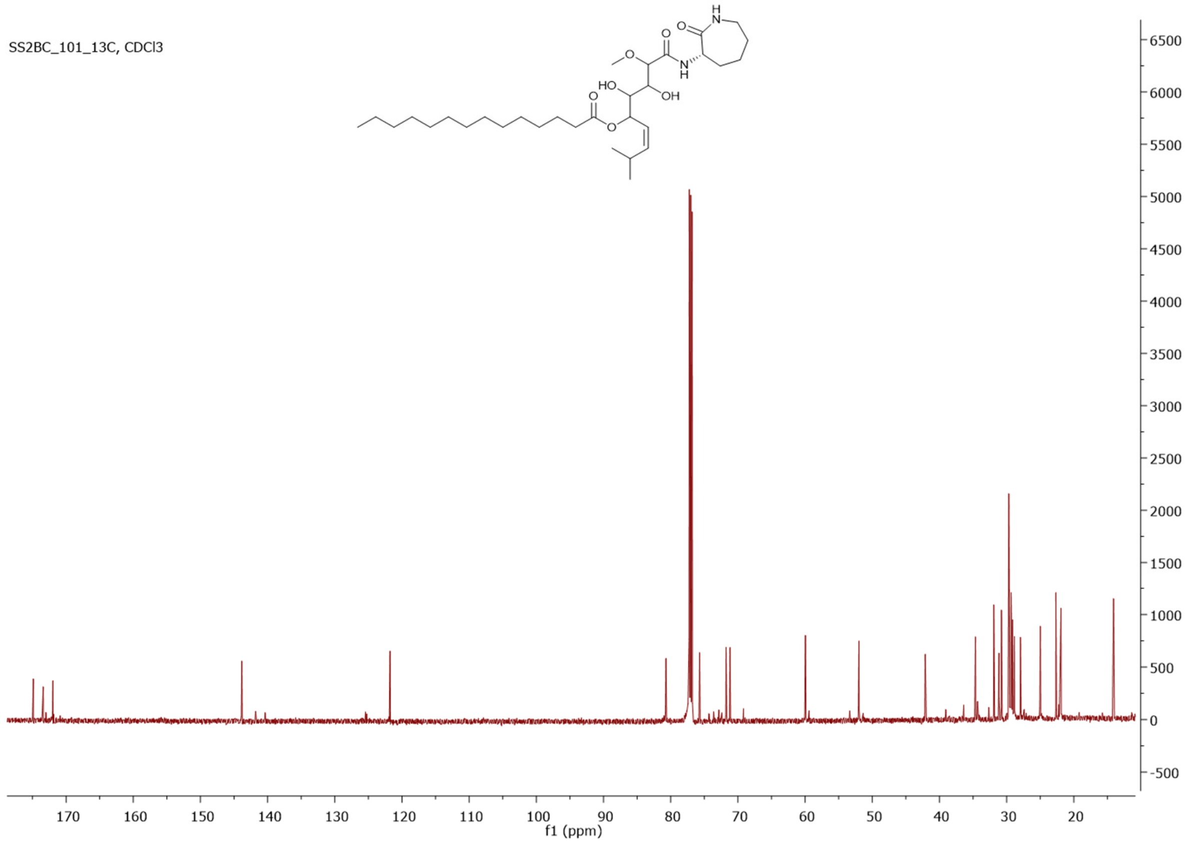

Figure S9. ^{13}C-NMR spectrum (150MHz, $CDCl_3$, 303K) of bengamide P **2**

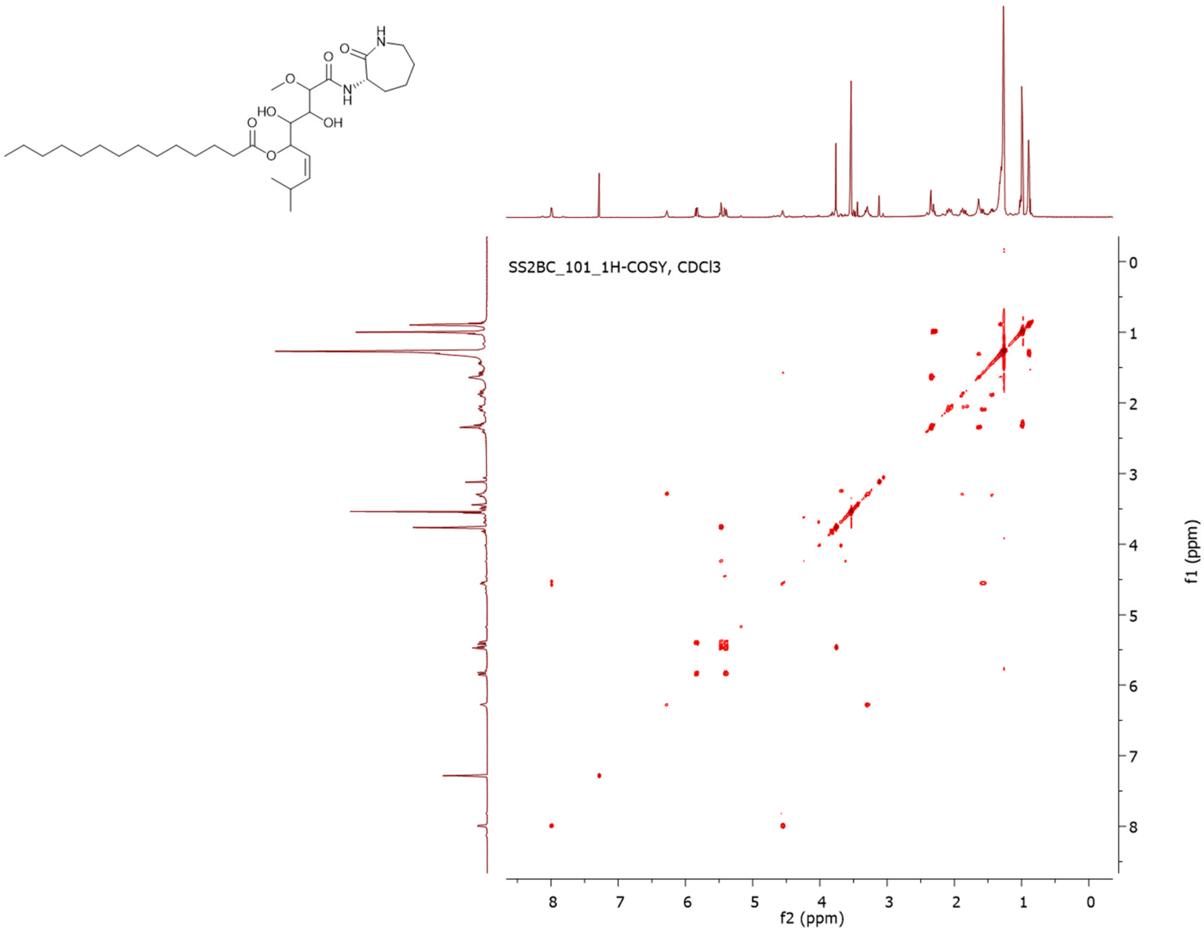

Figure S10. ^{1}H-^{1}H COSY spectrum (600MHz, $CDCl_3$, 303K) of bengamide P **2**

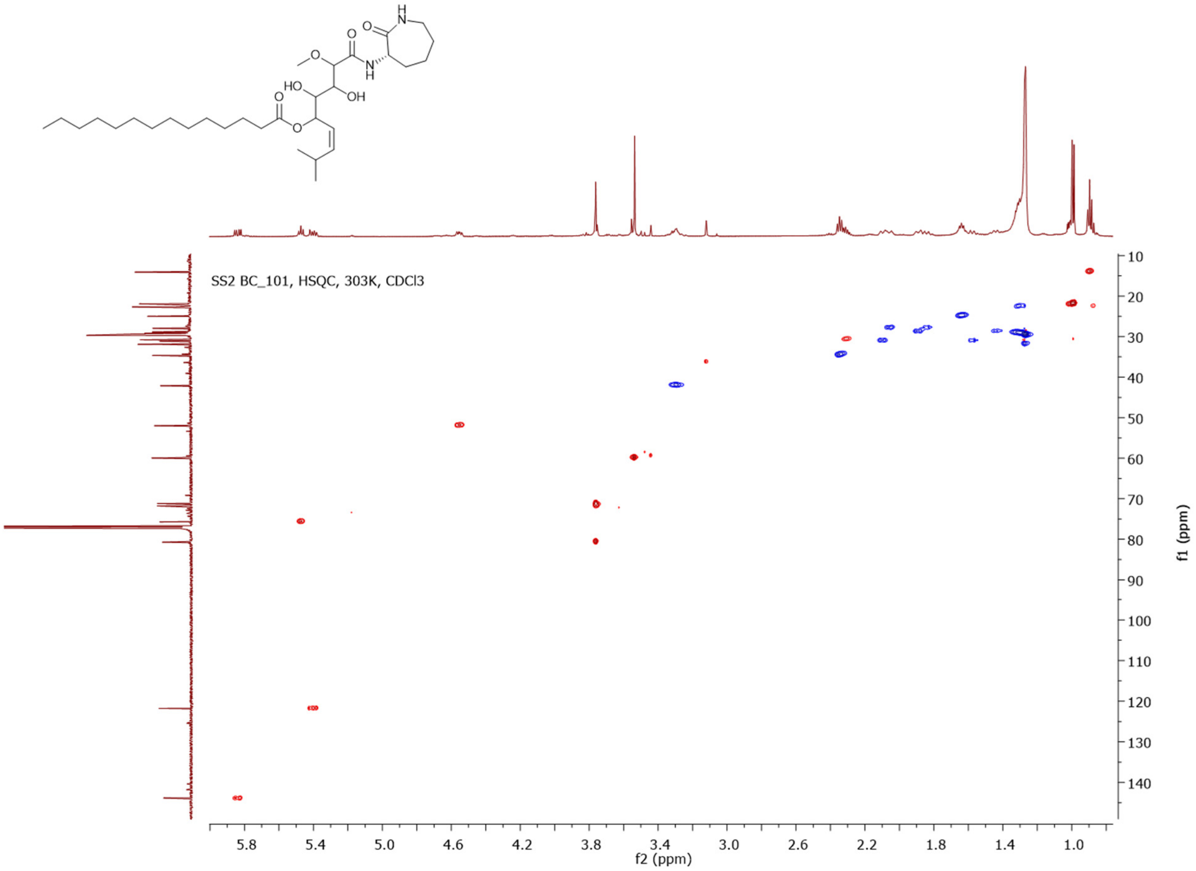

Figure S11. HSQC NMR spectrum (600MHz, $CDCl_3$, 303K) of bengamide P **2**

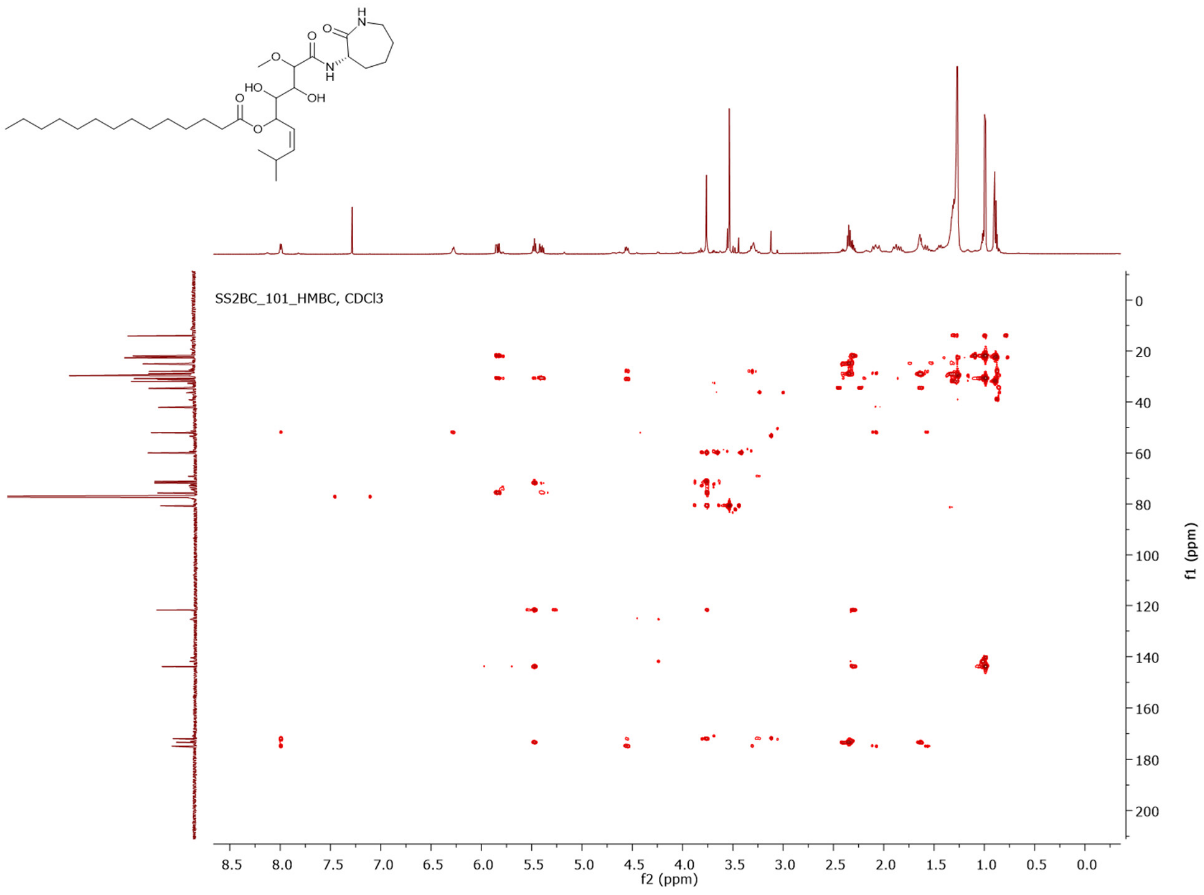

Figure S12. HMBC NMR spectrum (600MHz, $CDCl_3$, 303K) of bengamide P **2**

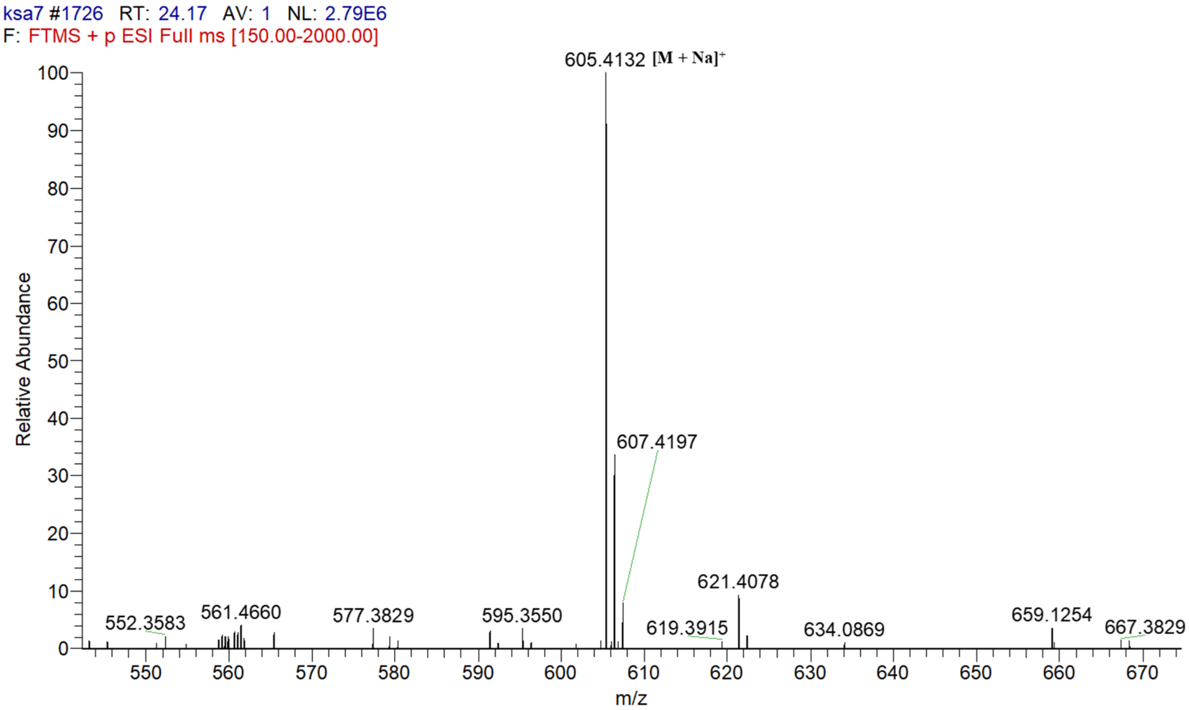

Figure S13. HR-ESI-MS spectrum of bengamide Q **3**

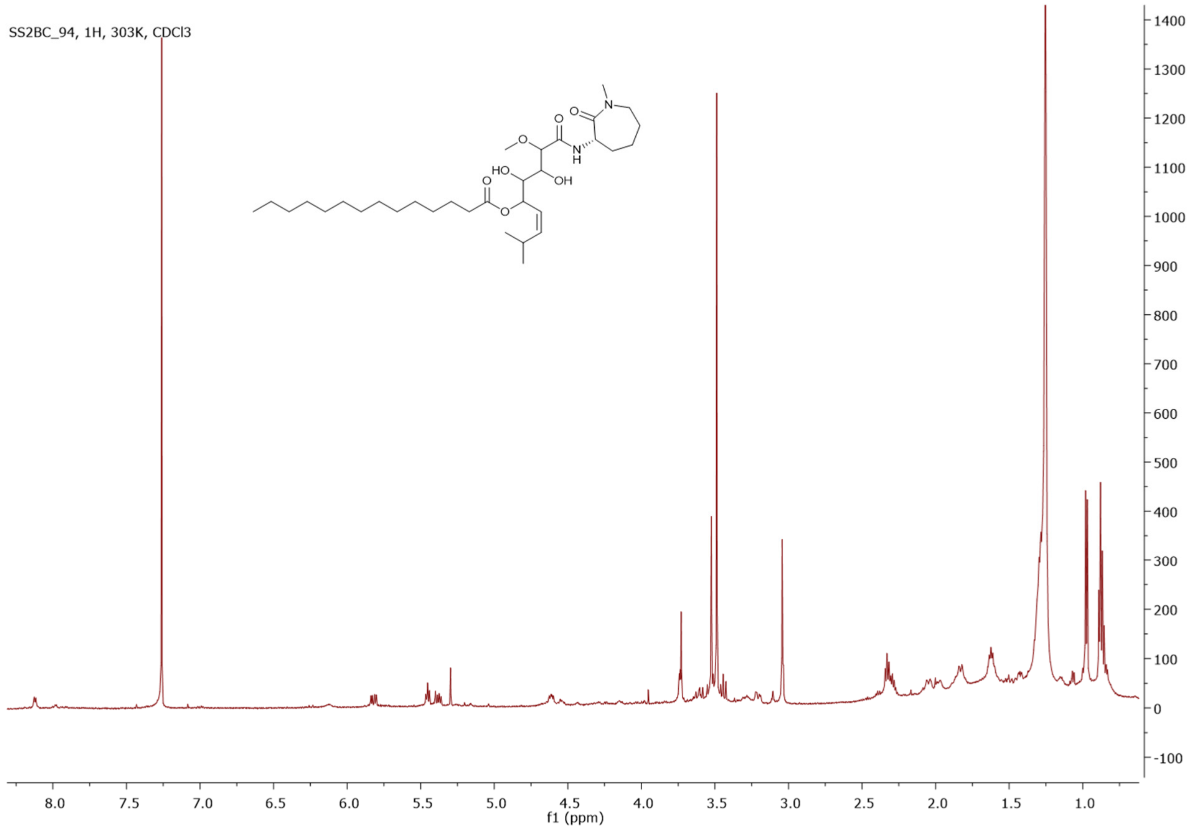

Figure S14. ^{1}H-NMR spectrum (600MHz, $CDCl_3$, 303K) of bengamide Q **3**

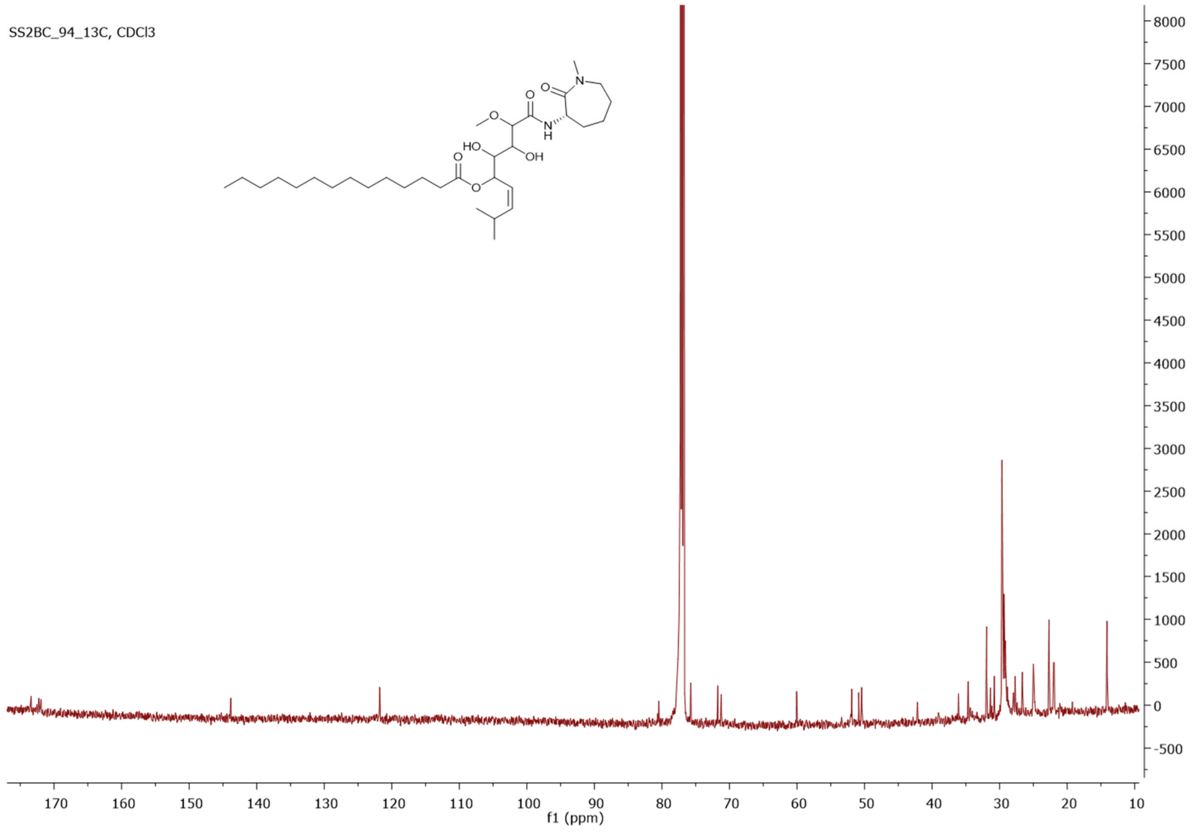

Figure S15. ^{13}C-NMR spectrum (150MHz, $CDCl_3$, 303K) of bengamide Q **3**

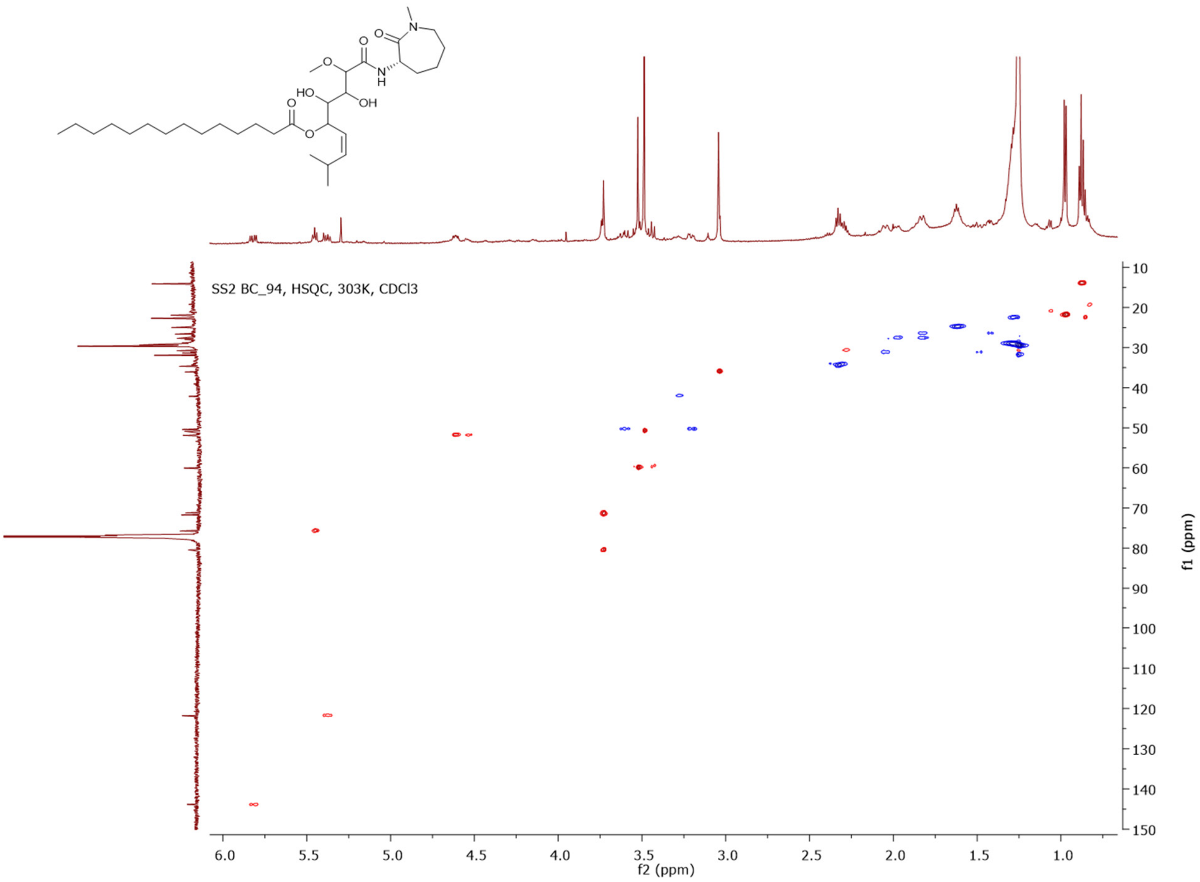

Figure S16. HSQC NMR spectrum (600MHz, $CDCl_3$, 303K) bengamide Q **3**

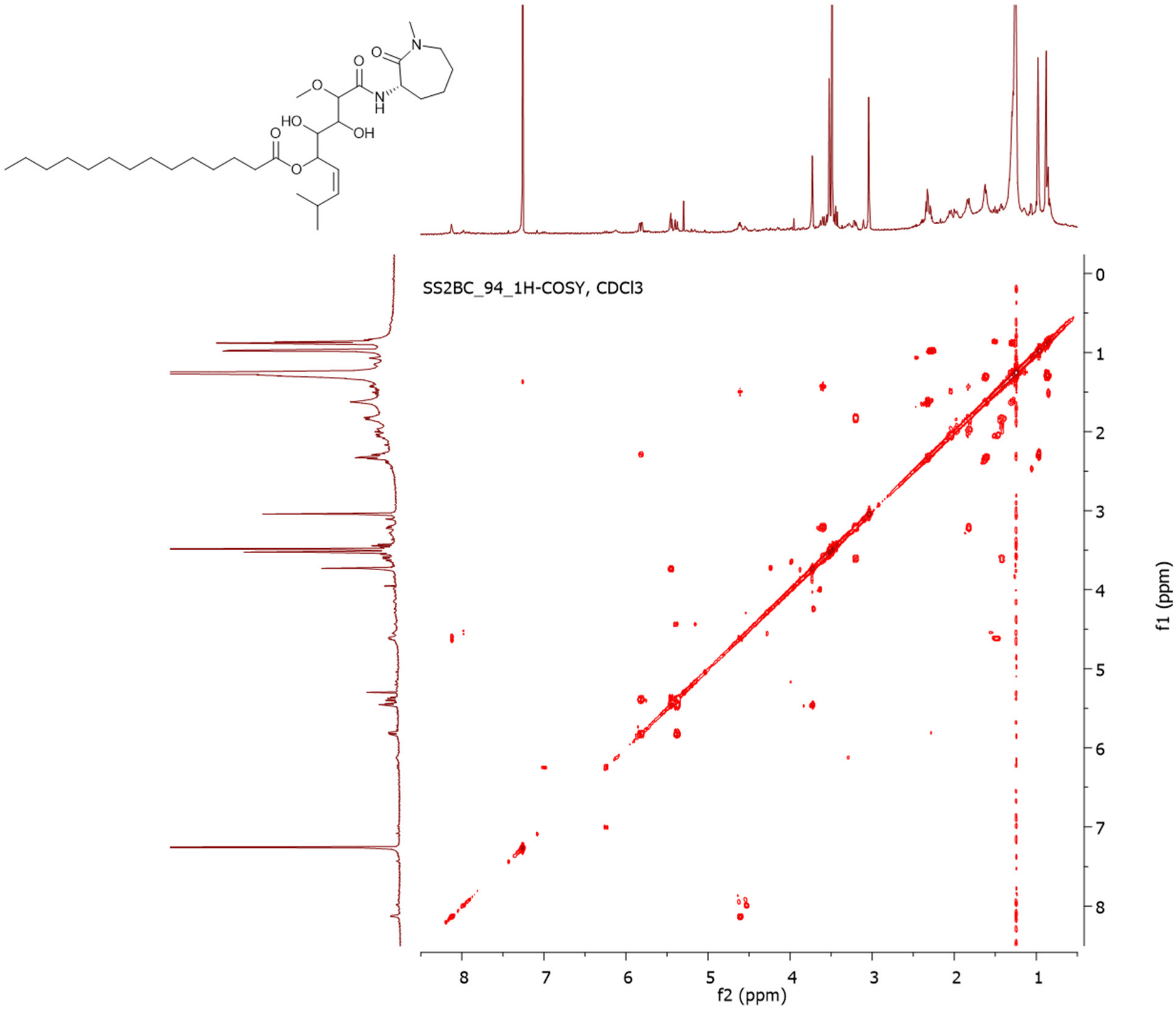

Figure S17. ^{1}H-^{1}H COSY spectrum (600MHz, $CDCl_3$, 303K) of bengamide Q **3**

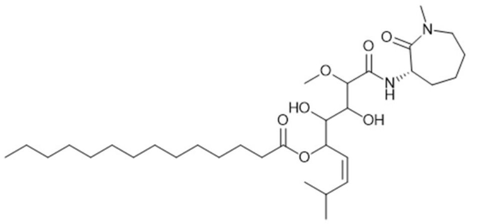

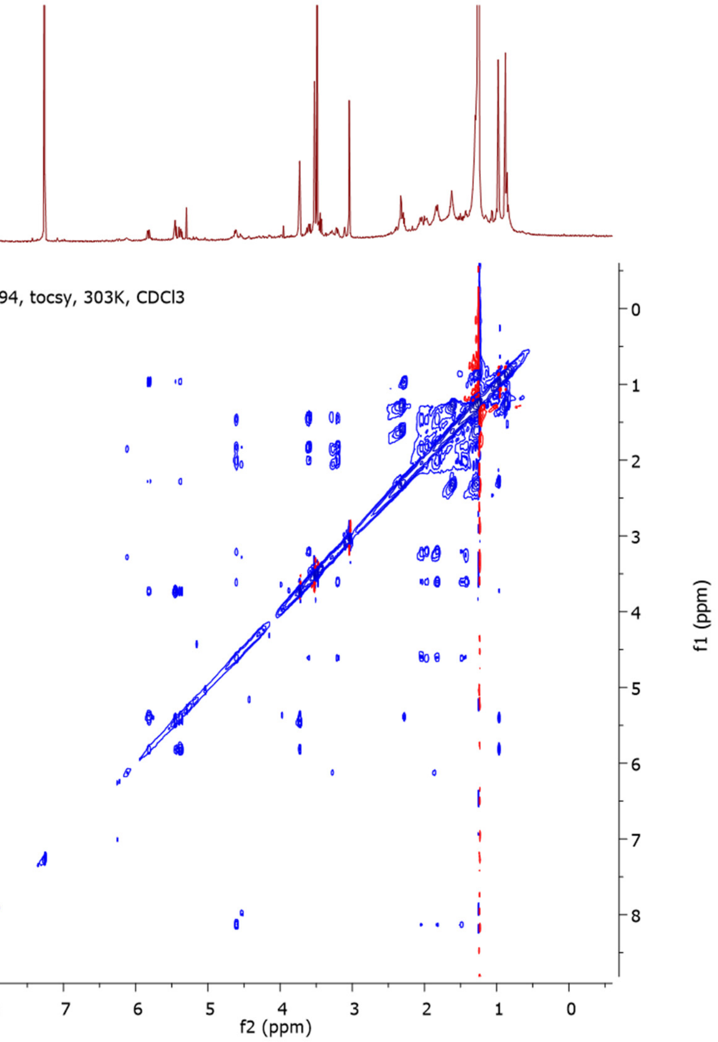

Figure S18. ^{1}H-^{1}H TOCSY spectrum (600MHz, $CDCl_3$, 303K) of bengamide Q **3**

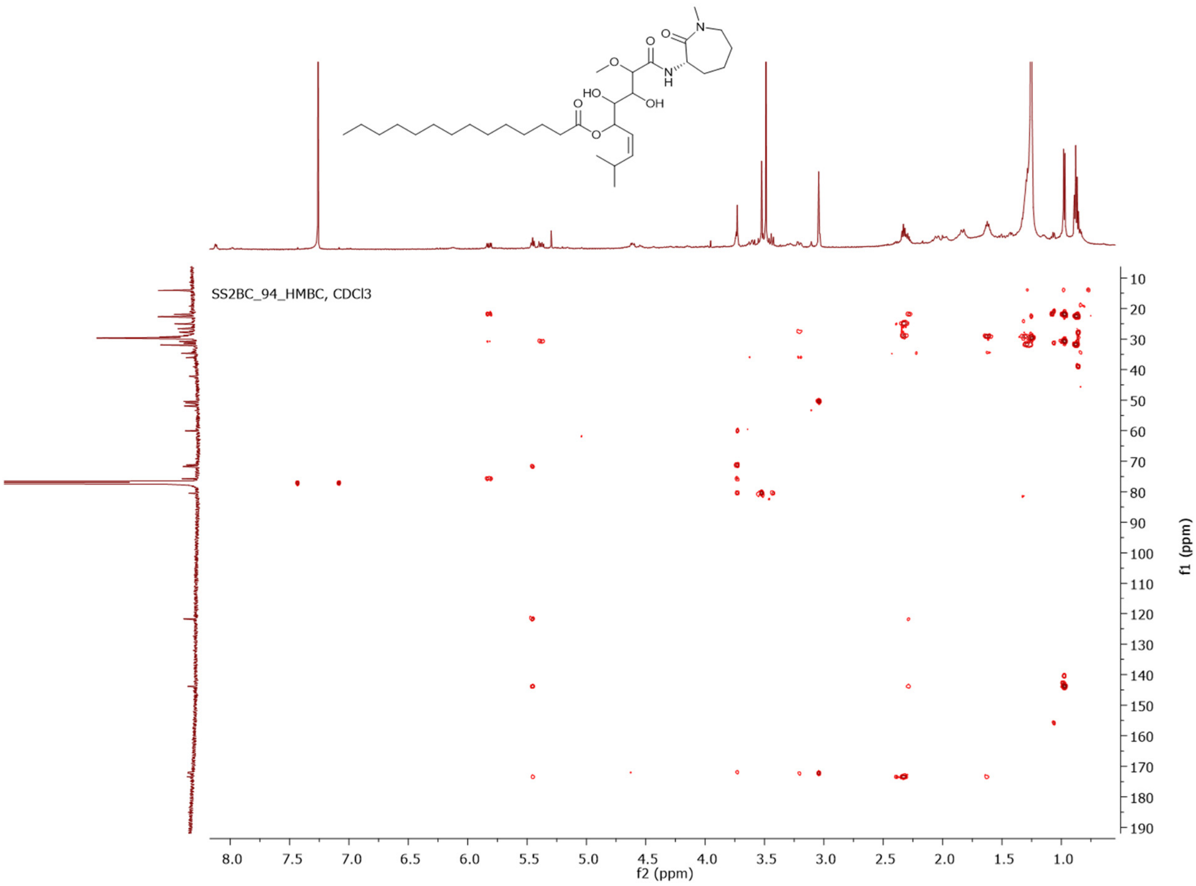

Figure S19. HMBC NMR spectrum (600MHz, $CDCl_3$, 303K) of bengamide Q **3**

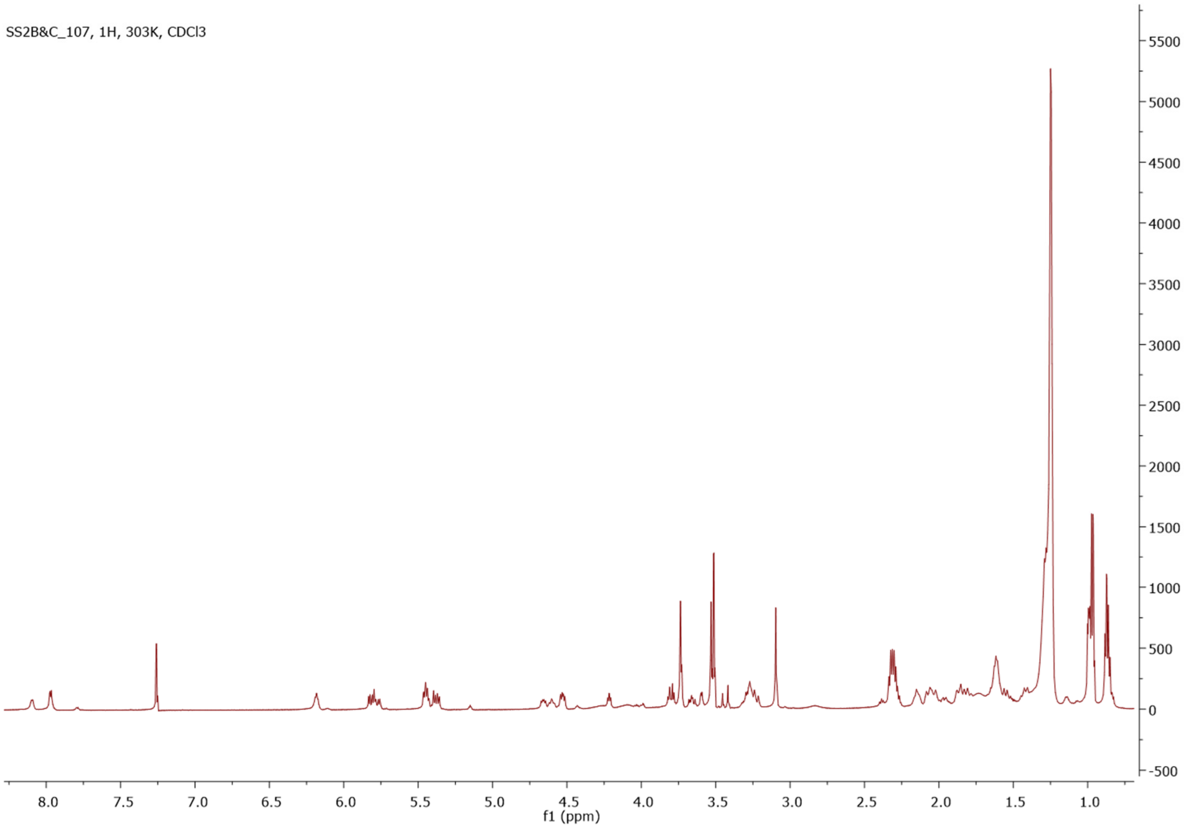

Figure S20. ^{1}H-NMR spectrum (600MHz, $CDCl_3$, 303K) of F107

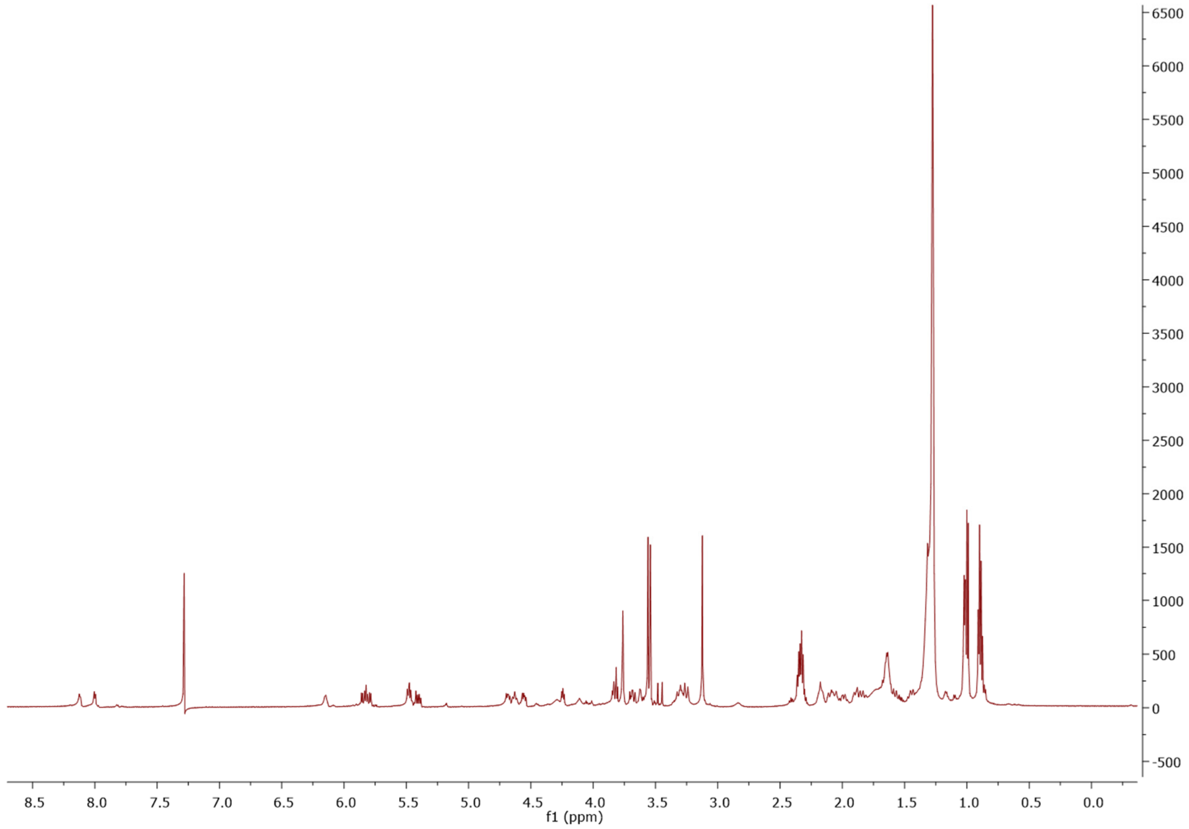

Figure S21. ^{1}H-NMR spectrum (600MHz, $CDCl_3$, 303K) of F114

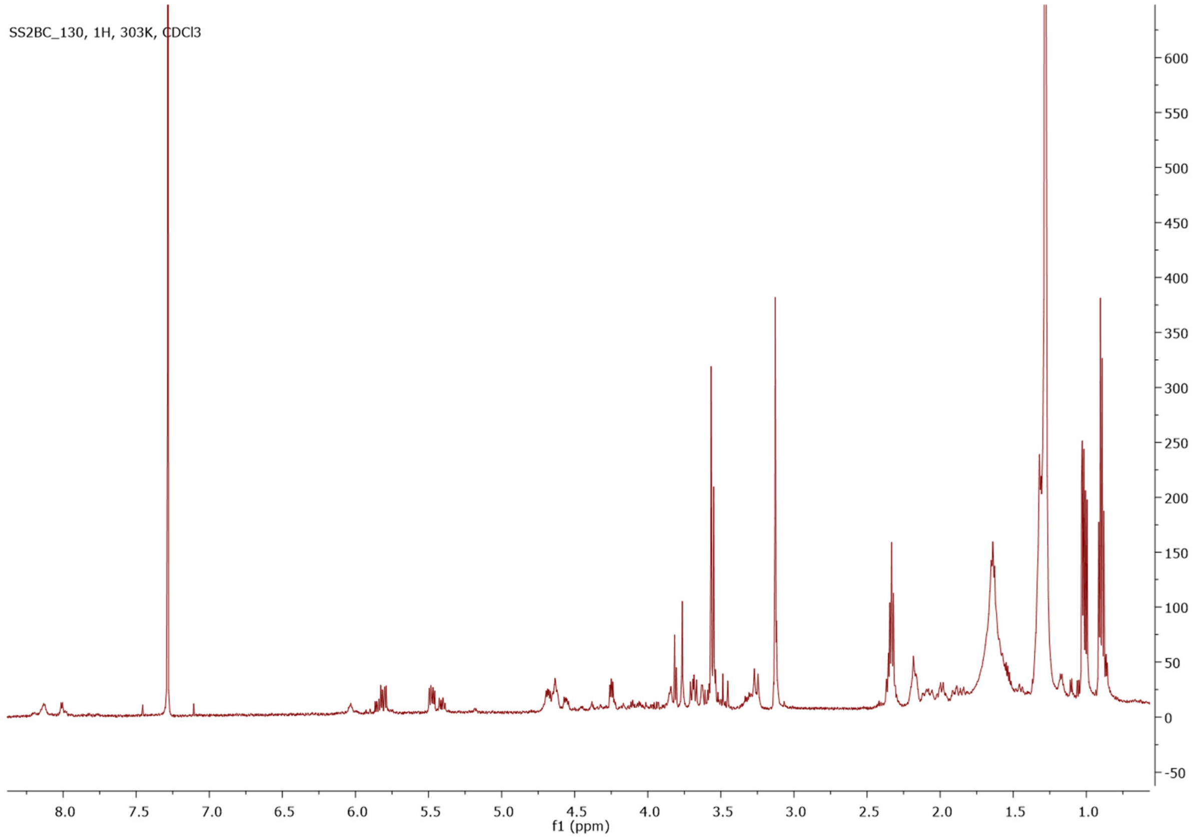

Figure S22. ^{1}H-NMR spectrum (600MHz, $CDCl_3$, 303K) of F130

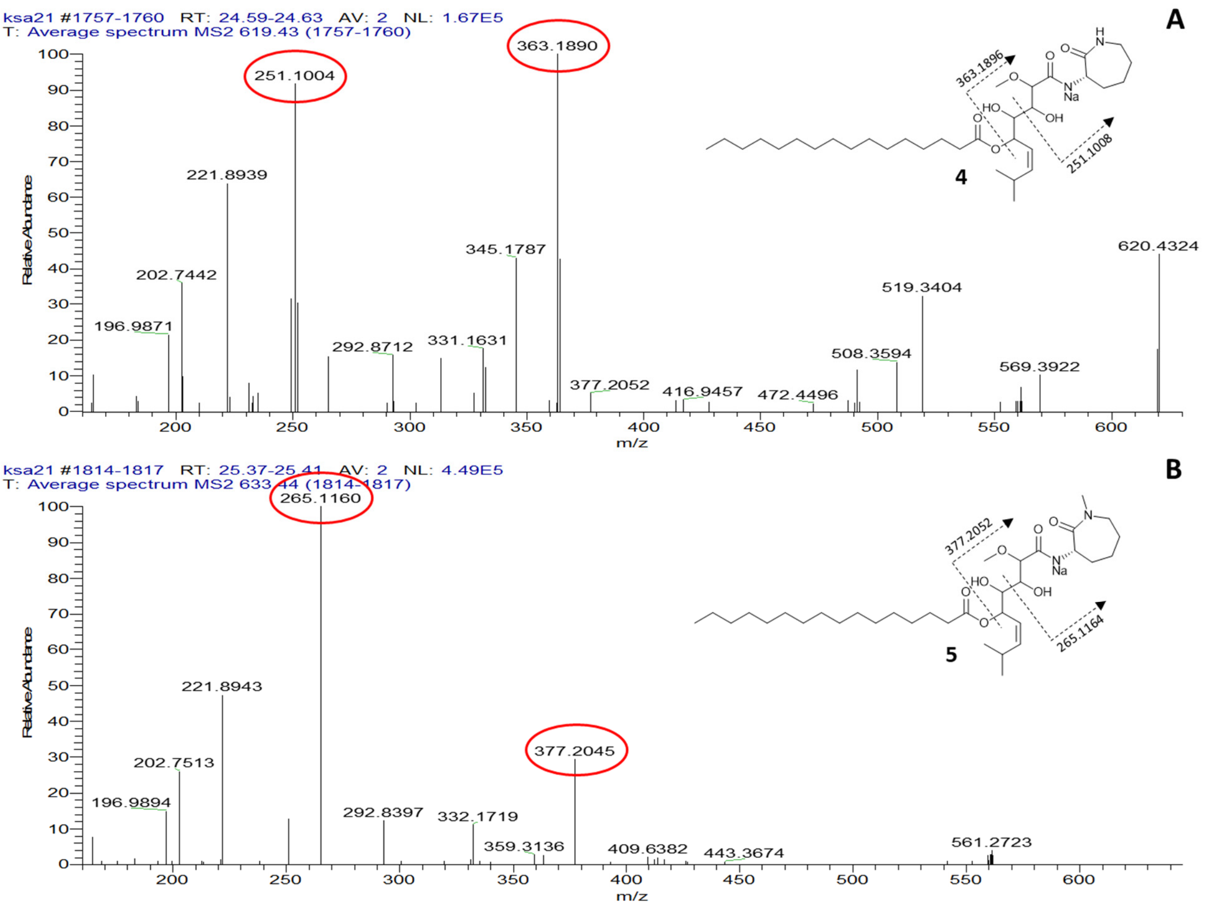

Figure S23. MS/MS fragmentation pattern of bengamide R **4** (A) and bengamide S **5** (B) with fragment ions circled in red showing methylation is on the nitrogen of the caprolactam ring.

Table S1. ^{13}C and ^{1}H chemical shifts of Heteronemin **1** isolated and that reported in literature.

	Isolated		Literature[1]	
Position	^{13}C	^{1}H, mult. (*J*, Hz)	^{13}C	^{1}H, mult. (*J*, Hz)
1	39.9	1.69<br>0.78	39.9	
2	18.2	1.53<br>1.36	18.2	
3	42.1	1.37 td J=4.03, 13.45, 13.51<br>1.11	42.0	
4	33.2		33.2	
5	56.5	0.78	56.5	
6	18.6	1.60<br>1.42	18.6	
7	41.9	1.73<br>0.91	41.8	
8	37.4		37.4	
9	58.8	0.78	58.8	
10	38.1		38.1	
11	27.2	1.69<br>1.46	27.2	
12	80.6	3.45 dd *J*=3.99, 11.37	80.5	3.44, dt, J =3.8.11.3
13	42.7		42.7	
14	54.8	0.92	54.7	
15	28.0	2.05<br>1.40	28.0	
16	69.4	5.36 dd J=6.06, 10.43	69.3	5.38 (dd,J = 4,10Hz)
17	114.4		114.4	
18	64.2	2.42	64.2	2.42
19	101.7	6.76 d *J*=1.38	101.6	6.77 (d, J= 1.3 Hz)
20	135.4	6.15 t *J*=1.93	135.3	6.17 (t, J = 2 Hz)
21	33.3	0.84	33.2	0.87
22	21.3	0.79	21.3	0.77
23	16.3	0.82	16.3	0.79
24	17.3	0.82	17.3	0.81
25	8.8	0.90	8.8	0.81
OAc	21.4	2.09	21.2	2.07
OAc	21.0	2.09	21.0	2.07
CO	171.3		171.3	
CO	170.1		170.1	

Table S2. ^{13}C and ^{1}H chemical shifts of bengamide P **2** isolated and that reported in literature.

	Isolated		Literature[2]	
Position	^{13}C	^{1}H, mult. (*J*, Hz)	^{13}C	^{1}H, mult. (*J*, Hz)
17	174.9		175.1	
16	173.3		173.6	
9	172.0		172.2	
3	143.8	5.84 (1H, dd, J= 6.47, 15.42 Hz)	144.1	5.83 (1H, dd, J= 6.5, 16.0 Hz)
4	121.8	5.40 (1H, dd, J= 7.84, 15.41 Hz)	122.0	5.39 (1H, dd, J= 7.5, 16.0 Hz)
8	80.7	3.76 (1H, bs)	80.8	3.74 (1H, bs)
5	75.7	5.47 (1H, t= 7.60 Hz)	75.9	5.47 (1H, t= 7.5 Hz)
7	71.8	3.75 (1H, bs)	72.0	3.74 (1H, bs)
6	71.2	3.75 (1H, bs)	71.4	3.74 (1H, bs)
13	28.8	1.88 (1H, m)<br>1.43 (1H, m)	29.1	1.86 (1H, m)<br>1.43 (1H, m)
OMe	60.0	3.53 (3H, s)	60.2	3.53 (3H, s)
10	52.0	4.55 (1H, m)	52.2	4.54 (1H, m)
14	42.1	3.29 (2H, m)	42.3	3.29 (2H, m)
18	34.6	2.34 (2H, t, J=7.32)	34.9	2.34 (2H, t, J=7.5)
12	27.9	2.05 (1H, m)<br>1.84 (1H, m)	28.2	2.07 (1H, m)<br>1.86 (1H, m)
28	31.9	1.27 (2H, m)	32.1	1.26 (2H, m)
2	30.9	2.30 (1H, m)	30.0	2.34 (1H, m)
20-27	30.8-29.1	1.27 (16H, m)	30.0-29.3	1.26 (16H, m)
11	31.1	2.09 (1H, m)<br>1.56 (1H, m)	31.4	2.07 (1H, m)<br>1.60 (1H, m)
19	25.0	1.64 (2H, m)	25.2	1.60 (2H, m)
29	22.8	1.28 (2H, m)	22.9	1.26 (2H, m)
1,15	21.9, 22.0	0.99 (6H, d J=6.88 Hz)	22.1,22.2	0.98 (6H, d J=7.0 Hz)
30	14.1	0.88 (3H, t J=7.08 Hz)	14.3	0.89 (3H, t J=7.0 Hz)

Table S3. ^{13}C and ^{1}H chemical shifts of bengamide Q **3** isolated and that reported in literature.

	Isolated		Literature[3]	
Position	^{13}C	^{1}H, mult. (J, Hz)	^{13}C	^{1}H, mult. (J, Hz)
17	173.4		173.6	
16	172.2		172.4	
9	171.9		172.1	
3	143.8	5.82 (1H, dd, J=6.47, 15.42 Hz)	144.0	5.83 (1H, dd, J=6.5, 16.0 Hz)
4	121.8	5.38 (1H, dd, J=7.92, 15.39 Hz)	122.0	5.39 (1H, dd, J=7.5, 16.0 Hz)
8	80.4	3.74 (1H, bs)	80.6	3.74 (1H, bs)
5	75.7	5.45 (1H, t, J=7.64 Hz)	75.9	5.46 (1H, t, J=7.5 Hz)
7	71.7	3.72 (1H, bs)	71.9	3.74 (1H, bs)
6	71.2	3.72 (1H, bs)	71.4	3.74 (1H, bs)
13	26.6	1.82 (1H, m)<br>1.42 (1H, m)	27.9	1.84 (1H, m)<br>1.47 (1H, m)
OMe	60.1	3.50 (3H, s)	60.3	3.54 (3H, s)
10	51.6	4.61 (1H, m)	50.6	4.62 (1H, m)
NMe	36.1	3.03 (3H, s)	36.3	3.05 (3H, s)
14	50.4	3.60 (1H, dd, J=15.50, 11.51)<br>3.21 (1H, dd, J=5.45, 15.53)	52.1	3.62 (1H, dd, J=15.5, 11.5)<br>3.22 (1H, dd, J=5.5, 15.5)
18	34.7	2.32 (2H, m)	34.9	2.34 (2H, t, J=7.5)
12	27.7	1.97 (1H, m)<br>1.82 (1H, m)	26.8	2.02 (1H, m)<br>1.84 (1H, m)
28	31.9	1.25 (2H, m)	32.1	1.26 (2H, m)
2	30.8	2.28 (1H, m)	31.0	2.34 (1H, m)
20-27	29.7-29.2	1.26 (16H, m)	29.9-29.3	1.26 (16H, m)
11	31.4	2.04 (1H, m)<br>1.49 (1H, m)	31.5	2.02 (1H, m)<br>1.63 (1H, m)
19	25.0	1.62 (2H, m)	25.2	1.63 (2H, m)
29	22.7	1.27 (2H, m)	22.9	1.26 (2H, m)
1,15	22.0, 21.9	0.97 (6H, d J=6.74 Hz)	22.2-22.1	0.98 (6H, d J=6.5 Hz)
30	14.3	0.87 (3H, t J=6.81 Hz)	14.3	0.89 (3H, t J=6.5 Hz)

Table S4. Compounds isolated and tentatively identified in the molecular cluster of the crude extract (SS2) of the sponge Jaspis splendens with their corresponding masses (observed and calculated), molecular formulae (MF), and mass error (ID (Δ ppm))

Compound	m/z ($[M+Na]^+$) Observed	m/z ($[M+Na]^+$) Calculated	MF	ID Δ ppm
Bengamide P	591.3978	591.3966	$C_{31}H_{56}N_2O_7$	2.03
Bengamide Q	605.4133	605.4122	$C_{32}H_{58}N_2O_7$	1.82
Bengamide A<br>Bengamide N<br>Bengamide H	607.3928	607.3915	$C_{31}H_{56}N_2O_8$	2.14
Bengamide R	619.4286	619.4279	$C_{33}H_{60}N_2O_7$	1.13
Bengamide B<br>Bengamide I<br>Bengamide L<br>Bengamide O	621.4084	621.4071	$C_{32}H_{59}N_2O_8$	2.09
Bengamide J<br>Bengamide M	635.4233	635.4228	$C_{33}H_{60}N_2O_8$	0.78
Bengamide S	633.4446	633.4455	$C_{34}H_{62}N_2O_7$	-1.42

References

1. Kazlauskas, R., Murphy,, P. T., Quinn, R. J., Wells, R. J. Heteronemin, a new Scalarin type Sesterterpene from the Sponge Heteronema erecta. *Tetrahedron Lett.* 1976, 2631–2634.

2. Bourguet-Kondracki, M. L., Martin, M. T., Debitus, C., Guyot, M. 12-epi-Heteroaemin : New Sesterterpene From Sponge the marine sponge Hyrtios erecta. *Tetrahedron Lett.* 1994, **35**, 109–110.

3. Thale, Z. *et al.* Bengamides Revisited : New Structures and Antitumor Studies. *J. Org. Chem.* 2001, 66, 1733–1741.
